# Ileocolic intussusception caused by ileal lipoma: A case report

**DOI:** 10.1097/MD.0000000000021525

**Published:** 2020-07-31

**Authors:** Chunyu Shi, Lu Pan, Bin Song, Yongjian Gao, Leichao Zhang, Ye Feng

**Affiliations:** a Department of Gastrointestinal Colorectal and Anal Surgery, China-Japan Union Hospital of Jilin University; b Department of Pediatric Immunology, Allergy and Rheumatology, The No.1 Hospital of Jilin University; c Department of Pathology, China-Japan Union Hospital of Jilin University, Changchun, China.

**Keywords:** intussusception, ileocolic, lipoma, adult

## Abstract

**Rationale:**

Adult intussusception is rarely observed, accounting for about 5% of all cases of intussusception. Most ileal lipomas are asymptomatic and do not need any special treatment. Herein, we describe a case with ileocolic intussusception caused by ileal lipoma.

**Patient concerns:**

A 27-year-old woman complaints of intermittent abdominal pain for 10 days.

**Diagnosis:**

Abdominal computed tomography demonstrated ileocolic intussusception. Colonoscopy revealed a spherical polypoid lesion with surface capillary rising from the lateral wall of the ileum. A diagnosis of ileocolic intussusception was made.

**Interventions:**

The patient underwent primary resection of the intussuscepted intestine after which an end-to-end anastomosis was performed.

**Outcomes:**

Histopathology report confirmed a 4.5 cm × 3.5 cm lipoma in the terminal ileum. The patient was discharged on a postoperative day 9 without complications.

**Lessons:**

We describe the difficulties in diagnosis and treatment of this rare cause of intussusception and review the literature on adult intussusceptions. The ileal lipoma is a very rare cause of ileocolic intussusception. Abdominal CT and colonoscopy are important for the diagnosis of intussusception and abdominal lipomas. Surgical resection remains the treatment of choice.

## 1 Introduction

Intussusception occurs when telescoping of a bowel segment into an adjacent distal segment. Adult intussusception is a rare cause of abdominal pain, accounting for only 1% to 5% of intestinal obstructions and 5% of intussusceptions.^[1]^ Compared with the intussusceptions in children, the clinical manifestations of adult intussuscepiton are nonspecific and chronic, therefore preoperative diagnosis remains difficult. Here, we describe a case of a 27-year-old female with ileocolic intussusception due to a lipoma arising from the ileum and resected by surgery.

## 2 Case presentation

**Figure 1:**
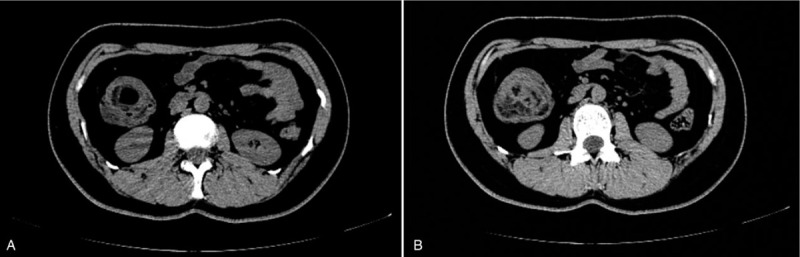
Abdominal computed tomography. (A) Abdominal CT showed ileocolonic intussusception; (B) Abdominal CT revealed fat density masses in ileocecus.

**Figure 2:**
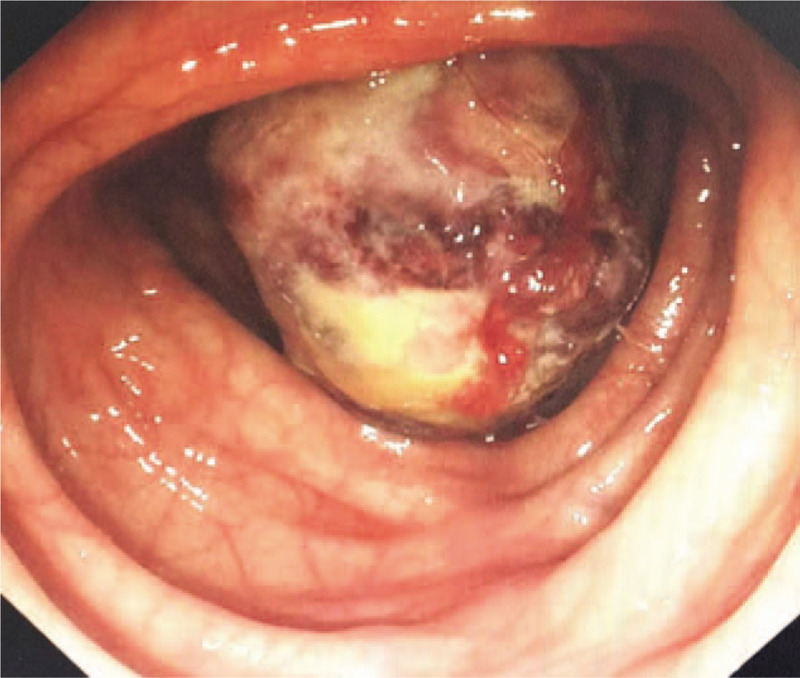
Colonoscopy showed an approximately 3.5 cm diameter spherical polypoid lesion near the ileocecal valve.

**Figure 3:**
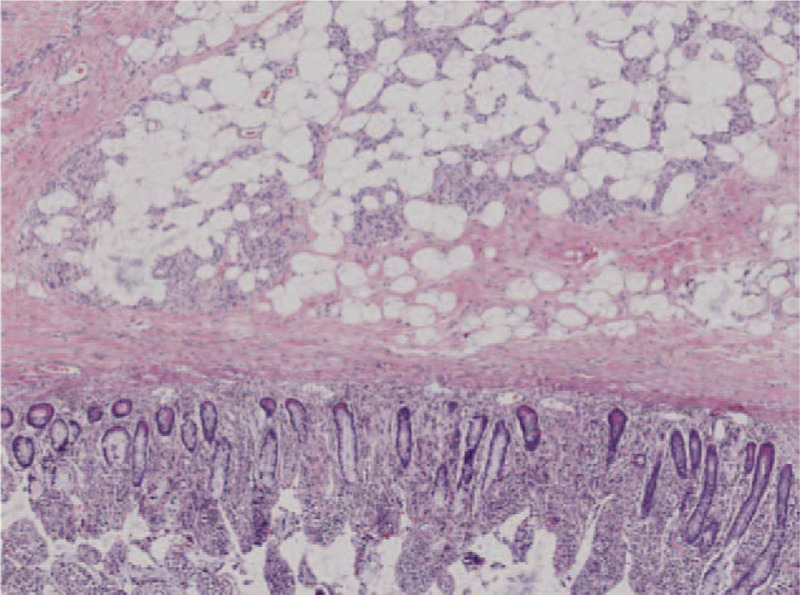
Histopathologic examination of the tumor revealed fat cells proliferating in the submucosal layer (hematoxylin and eosin, ×40).

A 27-year-old female was admitted to our department with a 10-day history of intermittent abdominal pain. The pain was moderate, paroxysmal, and colicky in nature, and it was present mainly in the right lower quadrant and radiated to nowhere. She denied any history of diarrhea, melena, hematochezia, weight loss, and bowel habit change. Physical examination revealed mild tenderness accompanied by a palpable mass in the right lower quadrant. The mass had a soft texture, unclear boundary, and a low degree of mobility. All of the laboratory studies were within normal limits. Abdominal computed tomography (CT) showed a target-like mass with fat density consistent with an ileocolic intussusception (Fig. 1). Colonoscopy revealed a 3.5 cm diameter, spherical polypoid lesion with surface capillary rising from the lateral wall of the ileum (Fig. 2). The patient was diagnosed with ileocolic intussusception with which was suspected to be a lipoma. Laparoscopic surgery was performed in December 2017. Laparotomy revealed the presence of an ileocolic intussusception caused by a mass in the terminal ileum. We performed primary resection of the intussuscepted intestine 20 cm proximal to the ileocecal valve. Approximately 15 cm of the small intestine, including the tumor, was resected after which an end-to-end anastomosis was performed. The pathological examination confirmed a diagnosis of lipoma (Fig. 3). The patient was discharged on the ninth postoperative day without complications. Until now, about two and a half years after surgery, he is in good condition and free of symptoms.

## 3 Discussion and conclusions

Adult intussusception is rarely observed compared with that in children, accounting for 5% of intussusception and ∼0.003% to 0.02% of all hospital admissions.^[1]^ Ninety-five percent of children intussusception are idiopathic with no structural lead points. However, 90% of adult intussusception have an organic cause, such as a benign polyp, enlarged mesenteric lymph node, lipoma, Meckel's diverticulum, lymphoma, gastrointestinal stromal tumor, primary, or metastatic adenocarcinoma.^[2]^ The exact mechanism is not clear. However, it is believed that any lesion in the bowel wall or irritant within the lumen that alters normal peristaltic activity can initiate an invagination.^[3,4]^ The most common site is the small bowel while the least common types are coloanal and gastroduodenal intussusceptions.^[5]^

The clinical manifestations of intussusceptions in adults are nonspecific and chronic. The typical pediatric triad, abdominal pain, palpable abdominal mass, and bloody stool are rarely seen in adults.^[7,8]^ Intermittent abdominal pain is the most common symptom followed by vomiting, nausea, and rectal bleeding.^[1,3,5]^ Abdominal masses are palpable in 24% to 42% of patients.^[6]^ Lipomas in gastrointestinal tract are generally asymptomatic and are usually incidentally found during colonoscopy, surgery. Because the symptoms are nonspecific, correct preoperative diagnosis of intussusception is difficult, with accuracy rates ranging from 30% to 90%.^[9,15,17,18]^ In a retrospective of 41 cases, 35% of the diagnosis is confirmed at laparotomy.^[19]^

Several methods can contribute to the preoperative diagnosis of intussusception, such as abdominal CT scan, abdominal ultrasound, plain film, angiography, and radionucleotide studies.^[6,10]^ Among them, Abdominal CT is reportedly the most useful tool. With typical target or sausage sign, abdominal CT scans are of great help of diagnosis of intussusception with diagnostic accuracy rates ranging from 58 to 100%.^[11–16]^ Moreover, abdominal CT has the advantages of revealing the site, level, and cause of intestinal obstructions.^[6]^ A colonoscopy helps evaluate intussusception, especially when the presenting symptoms indicate a large bowel obstruction.^[20]^ Whatsmore, colonoscopy helps in the identification of the lead point and pathologically diagnosis of intussusception. In our case, CT showed a target-like mass with fat density, and colonoscopy revealed a polyp that had prolapsed through the ileocecal valve. The diagnosis, although not pathologically determined, was ileocolic lipoma with intussusception.

In most infants and young children, barium enema is often used to diagnose and treat intussusception, however, it is rarely performed in adults. This is because of the high incidence of other pathologies associated with bowel intussusception in adults.^[21]^ Whatsmore, It is generally not advisable to reduce adult intussusceptions due to the possibility of bowel perforation and tumor cell dissemination. Hence, surgery is indicated in adult intussusceptions. The type of surgery differs and depends on the site, cause, and degree of obstruction. Most surgeons agree that resection is mandatory, especially in colonic intussusception and in patients more than 60 years old, because of the possibility of malignant tumor. Large bowel lesions were more frequently malignant than small bowel lesions. The incidence of malignancy associated with small bowel is less common than with the large bowel (31% vs 70%).^[5,22]^ For large bowel lesions on the right side, resection with primary anastomosis can be achieved in unprepared bowels. For left-sided or rectosigmoid lesions, resection with the construction of a colostomy and a Hartmann's pouch with reanastomosis at a second stage is considered safer.^[6]^ In our case, an ileal lipoma was suspected and ileocolic intussusception was diagnosed preoperatively. Because a benign lesion was highly suspected as the cause, we decided to perform radical right hemicolectomy.

Recently, there are several reports about laparoscopic resection in adult intussusception due to benign and malignant lesions of the small and large bowel.^[23–27]^ The choice of performing a laparoscopic or open approach based on the clinical condition of the patient, the location and extent of intussusception, the possibility of an underlying disease, and the availability of surgeons with sufficient laparoscopic expertise.^[27]^ In the present study, we did not use laparoscopy for diagnosis or treatment.

In conclusion, this is a case report of intussusception in an adult with an unusually benign cause, which was finally diagnosed with CT and colonoscopy and treated with surgical resection successfully.

## Acknowledgments

The authors thank the patient for his participation and his agreement to publication of the report.

## Author contributions

Chunyu Shi conceived the idea for this case report and performed the surgery with Ye Feng. Ye Feng performed the surgery. Leichao Zhang made the diagnosis. Bin Song and Yongjian Gao followed the patients with the author. LP helped modify the manuscript. All authors checked and approved the final manuscript.
